# *Staphylococcus epidermidis*—Skin friend or foe?

**DOI:** 10.1371/journal.ppat.1009026

**Published:** 2020-11-12

**Authors:** Morgan M. Brown, Alexander R. Horswill

**Affiliations:** 1 Department of Immunology and Microbiology, University of Colorado School of Medicine, Aurora, Colorado, United States of America; 2 Department of Veterans Affairs, Eastern Colorado Healthcare System, Aurora, Colorado, United States of America; Nanyang Technological University, SINGAPORE

## Abstract

Our skin is our first line of defense against environmental and pathogenic challenges. It is densely populated by a flora of bacteria, fungi, and viruses that normally interact with each other and with our immune system to promote skin health and homeostasis. *Staphylococcus epidermidis* is one of the most abundant bacterial colonizers of healthy human skin. While the field has historically assumed that all *S*. *epidermidis* isolates behave similarly, emerging evidence suggests that colonization by specific strains of *S*. *epidermidis* can either help or hurt the skin barrier depending on the context. In this short review, we discuss what is currently understood about *S*. *epidermidis* strain-level diversity and evaluate costs and benefits of *S*. *epidermidis* skin colonization. We challenge the current dogma that “all *S*. *epidermidis* strains behave equally” and posit that behavior is in fact highly context and strain dependent. Finally, in light of current proposals to use skin commensals as nonantibiotic treatments for acute or chronic skin diseases, we conclude that more work is urgently needed to fully understand the pathogenic and protective roles of commensals before we use them therapeutically.

## The human skin microbiota: Composition and function in barrier homeostasis

The human skin is a complex physiological barrier designed to maintain internal homeostasis and protect the host from opportunistic pathogens. The epidermis is composed of 4 stratified layers of terminally differentiating keratinocytes and studded with hair follicles and sebaceous glands. Taking these appendages into account, the surface area of the skin is at least 30 m^2^, which is even larger than the surface area of the gut [1]. The skin is densely populated by a diverse resident commensal flora of bacteria, archaea, fungi, and viruses [2]. Both metagenomics sequencing surveys and traditional culture methods have demonstrated that coagulase negative staphylococci (CoNS) are one of the most abundant colonizers of all skin sites [2]. The CoNS are a heterogeneous group of 38 species that are predominantly genetically and functionally uncharacterized. Despite this lack of characterization, there is mounting evidence that CoNS actively contribute to the maintenance of skin integrity and homeostasis by priming cutaneous immunity, controlling other resident flora, and preventing colonization by opportunistic pathogens (i.e., colonization resistance) [3]. Historically, the field has focused on the ubiquitous skin commensal *Staphylococcus epidermidis* with the assumption that all *S*. *epidermidis* strains or all CoNS behave similarly. In fact, current evidence suggests that *S*. *epidermidis* skin colonization may be far more nuanced and that colonization by specific strains of *S*. *epidermidis* may either help or hurt the skin barrier. Here, we discuss *S*. *epidermidis* strain-level diversity on skin, evaluate benefits and costs of *S*. *epidermidis* skin colonization, and comment on future directions in skin microbiome research with a focus on understanding how specific organisms, like *S*. *epidermidis*, contribute to skin health or disease.

## *S*. *epidermidis* strain-level diversity underlies skin–microbe interactions

*S*. *epidermidis* is by far the best studied member of the CoNS family and was historically used as a commensal comparator to its more pathogenic cousin, *Staphylococcus aureus* [4]. *S*. *epidermidis* can be been isolated from all skin microenvironments, including dry, moist, sebaceous, and foot regions [2]. However, the significant strain-level diversity among these isolates, especially specific virulence or host modulatory factors, is only beginning to be appreciated. Approximately 80% of the 2.5 Mb *S*. *epidermidis* genome is composed of core genes, whereas the remaining 20% is variable, indicating that *S*. *epidermidis* has an open pan-genome and a potentially unlimited genetic repertoire [5]. The observation that up to 20% of the genome can be exchanged with a larger pool of genes suggests that *S*. *epidermidis* is well poised to rapidly adapt to and thrive in all skin microenvironments. Indeed, a targeted metagenomics study revealed that there is an incredibly high spatiotemporal diversity of healthy skin *S*. *epidermidis* isolates between different skin microenvironments and between individuals [6]. Furthermore, these specialized communities are under high selective pressure, undergoing multiple horizontal gene transfer events via plasmid and phage to adapt to and persist in their specific skin niche [6].

One mechanistic example of the significance of *S*. *epidermidis* strain-level diversity and its implications for overall skin health is the accessory gene regulator (*agr*) quorum sensing system. The *agr* locus (*agrBDCA*) is conserved across all staphylococci, including *S*. *epidermidis*. The *S*. *epidermidis agr* regulon controls production of a small suite of potential virulence factors like proteases, lipases, and immunomodulatory phenol soluble modulins (PSMs), and retention of the *agr* system is necessary for skin colonization [7]. Importantly, every *S*. *epidermidis* strain is a single *agr* type (I–IV) determined by a hypervariable region spanning *agrBDC* [6,7]. While most individuals are dominantly colonized by a single *S*. *epidermidis agr* type, minor subpopulations of nondominant *agr* types in specific skin sites are also common [6]. Certain *S*. *epidermidis agr* types, as well as other CoNS species, make small peptides that inhibit noncognate *S*. *epidermidis agr* signaling [7,8]. This observation suggests that *agr* heterogeneity in concert with total CoNS diversity may be an important factor in promoting homeostatic *S*. *epidermidis* skin colonization and suppressing virulence factor production [6–8]. As *agr* activity is controlled by bacterial density, low absolute numbers of *S*. *epidermidis* on the skin may also contribute to low *agr* activity under homeostatic conditions [6,7]. Future work could address these inter- and intraspecies *agr* interactions and their implications for skin health in larger patient cohorts. Furthermore, *S*. *epidermidis agr* is only 1 example of how strain variation may have specific functional outcomes for skin health. Metagenomics analyses to reveal which strains are present, coupled with mechanistic studies to understand strain-level functionality, will likely uncover more intriguing links between *S*. *epidermidis* strain-level diversity and skin health status.

## Benefits of *S*. *epidermidis* skin colonization

**Fig 1:**
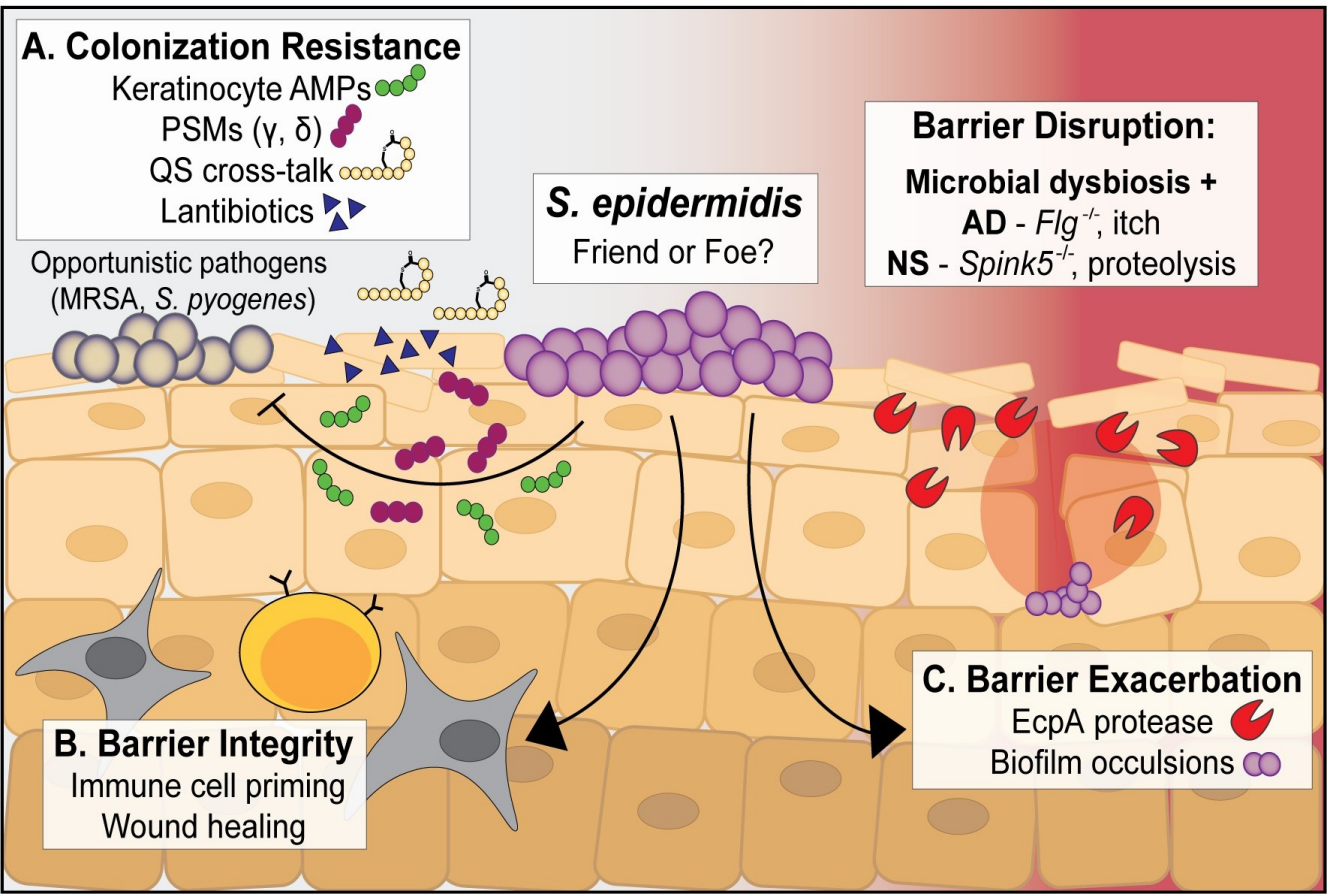
The ubiquitous skin commensal *S*. *epidermidis* positively and negatively impacts barrier homeostasis and integrity. **(A)** *S*. *epidermidis* phenol soluble modulins PSM*γ* and PSM*δ* can synergize with keratinocyte-derived AMPs to kill opportunistic skin pathogens like MRSA and *Streptococcus pyogenes*. *S*. *epidermidis* also makes anti-MRSA quorum sensing inhibitor peptides and a variety of small antimicrobials known as lantibiotics to mediate skin colonization resistance. **(B)** *S*. *epidermidis* lipoteichoic acid and some lipopeptides can dampen the inflammatory response to skin injury, accelerating wound healing. Early skin colonization with *S*. *epidermidis* is crucial for development of immune cell subsets including effector T cells and MAIT cells, and long-term *S*. *epidermidis* skin colonization may help the cutaneous immune system distinguish between commensal and pathogenic bacteria. **(C)** Certain *S*. *epidermidis* strains can “bloom” and exacerbate AD or NS skin lesions through production of the EcpA protease. Inflammatory *S*. *epidermidis* biofilms that occlude sweat glands have also been shown to exacerbate some AD lesions. AD, atopic dermatitis; AMPs, antimicrobial peptides; MAIT cells, mucosal-associated invariant T cells; MRSA, methicillin-resistant *Staphylococcus aureus*; NS, Netherton syndrome; PSM, phenol soluble modulins; QS, quorum sensing.

Significant attention in the field has been given to *S*. *epidermidis* and its role as a beneficial skin commensal (Fig 1). For example, *S*. *epidermidis* activated distinct innate immune signaling pathways in human keratinocytes to augment antimicrobial peptide (AMP)-mediated killing of *S*. *aureus*, though the secreted factor necessary for this enhancement was not elucidated [9]. *S*. *epidermidis* PSMs are small, amphipathic *α*-helical peptides that are abundantly produced on normal epidermis and in hair follicles [10]. PSMs synergized with host AMPs to enhance killing of the pathogen *Streptococcus pyogenes* [10]. In a mouse model of skin injury, both *S*. *epidermidis* lipoteichoic acid [11] and the lipopeptide LP78 [12] attenuated the inflammatory response to accelerate wound healing in a Toll-like receptor (TLR)-3-dependent mechanism. Finally, some strains of *S*. *epidermidis* can dampen *S*. *aureus*–induced neutrophil recruitment and pro-inflammatory cytokine production, which could potentially be protective against more severe skin infection [13].

In addition to modulating the innate immune response to skin infection or damage, *S*. *epidermidis* colonization contributes to the development and priming of the adaptive immune system. Studies of gnotobiotic mice revealed that *S*. *epidermidis* skin colonization is necessary for effector T cell development and function [14] as well as early localization and priming of mucosal-associated invariant T cells (MAIT cells), which are an important component of nonclassical cutaneous immune signaling that mediates distinct patterns of host–commensal cross talk [15]. The skin is also home to one of the largest reservoirs of effector T cell subsets, and there is growing appreciation of the depth and complexity of cross talk between these tissue-resident lymphocytes and colonizers like *S*. *epidermidis* [16]. Taken together, *S*. *epidermidis* is undoubtedly important for priming innate and adaptive defenses against pathogens and promoting homeostasis. However, future work may reveal that other CoNS, in concert with *S*. *epidermidis*, contribute more substantially to the full picture of skin development and health than previously appreciated.

## Costs of *S*. *epidermidis* skin colonization

While widely appreciated as an abundant skin symbiont, emerging evidence suggests that skin colonization by specific strains of *S*. *epidermidis* may actually be detrimental to the host under certain conditions. The intact skin is a formidable barrier to pathogens and commensals alike, but disruption of this barrier, through either genetic mutation or physical disruption, can dramatically alter *S*. *epidermidis* behavior from benign to pathogenic (Fig 1). For example, murine skin pretreated with *S*. *epidermidis* was only resistant to *S*. *aureus* challenge when the barrier was intact, not when it was physically disrupted by tape stripping prior to bacterial inoculation [17]. In atopic dermatitis (AD, i.e., eczema), patients are often highly colonized with *S*. *aureus* at lesional sites, and this bacterial “bloom” positively correlates with disease severity [18]. Longitudinal metagenomics studies have shown that some AD patients can be highly colonized by *S*. *epidermidis* rather than *S*. *aureus* at lesional sites. It has been postulated that such outgrowth may similarly correlate with disease severity; however, there have been few investigations of the mechanistic basis of *S*. *epidermidis*–mediated AD barrier exacerbation [19,20].

Recently, the cysteine protease EcpA was identified as a key mediator of *S*. *epidermidis*–induced AD barrier degradation [8,21]. Underscoring the importance of strain-level diversity, EcpA is present in all *S*. *epidermidis* strains but only seems to be expressed by a subset [8]. EcpA has significant sequence similarity and protein homology to the well-characterized *S*. *aureus* staphopains A and B, which can digest the AMP LL-37 to enhance *S*. *aureus* biofilm growth in AD lesions [21,22]. EcpA degraded multiple components of the skin barrier, including LL-37 as well as desmoglein-1, and significantly contributed to increased inflammation and barrier dysfunction in mouse models of AD [8]. Aside from AD, *S*. *epidermidis* overexpansion and EcpA production are also linked to exacerbation of Netherton syndrome (NS), a skin disorder characterized by high levels of serine protease activity caused by a mutation in the gene *SPINK5* [21]. Importantly, EcpA production is regulated by the *S*. *epidermidis agr* quorum sensing system [7]. These observations suggest a possible mechanism of *S*. *epidermidis* exacerbation of AD and NS, where there is some initial dysbiosis of inhibitory *S*. *epidermidis* or CoNS strains, followed by deinhibition of *S*. *epidermidis agr* signaling. This would facilitate the outgrowth of 1 *S*. *epidermidis agr* type (most commonly *agr*-I) [23] and the up-regulation of virulence factors like EcpA [8]. Such enhanced expression of EcpA and other virulence factors, combined with genetic or environmental barrier disruption in both skin diseases, would provide an ideal environment for *S*. *epidermidis* expansion and exacerbation. Finally, the propensity of *S*. *epidermidis* to form biofilms may also exacerbate AD, as inflammatory biofilm communities of both *S*. *aureus* and *S*. *epidermidis* have been documented in some sweat glands at AD lesional sites [24]. However, it is still unclear to what extent *S*. *epidermidis* biofilms form on normal or diseased skin, and more work is needed to fully understand the impact of biofilms in AD or other skin diseases.

## Concluding remarks and future directions

Together, these findings demonstrate the variable physiology and contextual control of *S*. *epidermidis* on skin and underscore the potential duality of the *S*. *epidermidis* lifestyle as colonizer or pathogen. Future work should continue to evaluate (with strain-level resolution) how this complex organism fits into the larger context of skin health. While the field has rapidly shifted to metagenomics analysis of “who’s there” on skin during health or disease, it is imperative to continue to define and understand the specific mechanisms that regulate commensal colonization as well as pathogenicity. This is especially true for the growing demands to utilize commensal bacteria as nonantibiotic treatments for skin diseases such as AD. While there is some successful precedence for using CoNS as an anti-methicillin–resistant *S*. *aureus* (MRSA) topical treatment [25], it is imperative to fully appreciate and regulate an organism’s potential for pathogenicity (i.e., *S*. *epidermidis* EcpA production) before widespread use as a therapeutic. As for other skin-dominant CoNS like *Staphylococcus warneri*, *Staphylococcus hominis*, and *Staphylococcus capitis*, their roles in colonization resistance or their potential for pathogenicity are even less well defined than *S*. *epidermidis*. These highly abundant yet understudied CoNS species, in addition to non-staphylococcal members of the microbiota like *Corynebacterium* spp. or *Cutibacterium* spp., represent a potential wealth of mechanistic information on interactions between the microbiota, host epithelia, and opportunistic pathogens that remain to be discovered. In conclusion, we posit that this high-resolution understanding of skin commensals, with an emphasis on benefits and costs of colonization, will fundamentally alter how we manage or treat our skin health.
